# Enhanced efficiency of generating human-induced pluripotent stem cells using Lin28-30Kc19 fusion protein

**DOI:** 10.3389/fbioe.2022.911614

**Published:** 2022-07-22

**Authors:** Boram Son, Hyungro Yoon, Jina Ryu, Haein Lee, Jinmyoung Joo, Hee Ho Park, Tai Hyun Park

**Affiliations:** ^1^ Department of Bioengineering, Hanyang University, Seoul, South Korea; ^2^ Interdisciplinary Program in Bioengineering, Seoul National University, Seoul, South Korea; ^3^ School of Chemical and Biological Engineering, Institute of Chemical Processes, Seoul National University, Seoul, South Korea; ^4^ Department of Biomedical Engineering, Ulsan National Institute of Science and Technology (UNIST), Ulsan, South Korea; ^5^ Education and Research Group for Biopharmaceutical Innovation Leader, Hanyang University, Seoul, South Korea; ^6^ BioMAX/N-Bio Institute, Institute of Bioengineering, Seoul National University, Seoul, South Korea

**Keywords:** human induced pluripotent stem cells (hiPSCs), 30Kc19, Lin28, fusion protein, soluble

## Abstract

Induced pluripotent stem cells (iPSCs) have intrinsic properties, such as self-renewal ability and pluripotency, which are also shown in embryonic stem cells (ESCs). The challenge of improving the iPSC generation efficiency has been an important issue and there have been many attempts to develop iPSC generation methods. In this research, we added Lin28, known as one of the reprogramming factors, in the form of a soluble recombinant protein from *E. coli* to improve the efficiency of human iPSC (hiPSC) generation, in respect of alkaline phosphatase (AP)-positive colonies. To deliver Lin28 inside the cells, we generated a soluble Lin28-30Kc19 fusion protein, with 30Kc19 at the C-terminal domain of Lin28. 30Kc19, a silkworm hemolymph-derived protein, was fused due to its cell-penetrating and protein-stabilizing properties. The Lin28-30Kc19 was treated to human dermal fibroblasts (HDFs), in combination with four defined reprogramming factors (Oct4, Sox2, c-Myc, and Klf4). After 14 days of cell culture, we confirmed the generated hiPSCs through AP staining. According to the results, the addition of Lin28-30Kc19 increased the number and size of generated AP-positive hiPSC colonies. Through this research, we anticipate that this recombinant protein would be a valuable material for increasing the efficiency of hiPSC generation and for enhancing the possibility as a substitute of the conventional method.

## Introduction

In the field of regenerative medicine, embryonic stem cells (ESCs) have emerged as a novel material because of their unique characteristics represented as pluripotency, also known as the potential of differentiation (McCulloch and Till, 2005). However, ethical issues are raised because ESCs are isolated from fertilized eggs during embryogenesis (Robertson, 2001). To overcome this kind of limitation, different types of cells, known as induced pluripotent stem cells (iPSCs), were generated and then suggested as an alternative for the ESCs (Takahashi et al., 2007). The iPSCs derived from a patient’s somatic cells are expected to be utilized for disease modeling and the selection of appropriate drug candidates. By using the iPSCs, it would be possible to verify the efficacy and stability of a new drug candidate in the pre-clinical stage. In addition, it is a technology with great potential that can be applied to the production of artificial tissues and organs, used for tissue regeneration and organ transplantation. Therefore, the use of iPSCs has become a significant source for personalized medical care.

Despite those advantages in the application of iPSCs, there have been several limitations by technical difficulties. Due to the low efficiency of iPSC generation, there have been many efforts to enhance the generation efficiency. According to recent studies, in addition to the four transcription factors conventionally used for the reprogramming (octamer-binding transcription factor 4; Oct4, sex-determining region Y-box 2; Sox2, cellular myelocytomatosis; c-Myc and kruppel-like factor 4; also Klf4, also known as Yamanaka factors) (Nakagawa et al., 2008), other transcriptional factors named “fifth factor” were complemented; one of them, RNA-binding protein Lin28 increased the efficiency of iPSC production (Yu et al., 2007). In other studies, the four reprogramming factors were converted into recombinant proteins in the form of binding to a cell-permeable protein, in order to diminish the risk of viral deoxyribonucleic acid (DNA) integration (Kim et al., 2009; Zhou et al., 2009). Although the reprogramming, relying on the transcription factors in the form of recombinant proteins, insisted an advance in iPSC generation in respect of risk reduction in the permanent integration of DNA, it was challenging to produce soluble recombinant proteins for reprogramming. The drawbacks of the protein-based approaches were inclusion body forming-reprogramming factors in the microbial expression system and low production in the mammalian system. In order to overcome those limitations in recombinant protein production, reprogramming factors were expressed as cell transactivator of transcription (TAT) fusion proteins in baculovirus-infected Sf9 insect cells (Pan et al., 2015). However, according to this study, none of these recombinant proteins were expressed as soluble and secreted forms. All the TAT-fused reprogramming factors, including Lin28, were detected only in the cell debris (Pan et al., 2015). This inclusion body-formation characteristic of the reprogramming proteins makes the purification process difficult and limits their use for cellular reprogramming. Based on these results, in this study, we tried to enhance the production efficiency of hiPSCs free from a problem of viral gene integration, by mass production and the addition of a soluble form of transcription factor Lin28.

30Kc19 protein is present in the hemolymph of silkworms, *Bombyx mori*, and it is a member of the 30K protein group (Izumi et al., 1981; Sakai et al., 1988). Previously, it has been shown that the 30Kc19 protein has an anti-apoptotic effect when treated in the medium of insect cells and animal cells (Rhee et al., 2009). In addition, it was also confirmed that the 30Kc19 protein can maintain a high mitochondrial membrane potential of cells, and thus increase ATP production (Park et al., 2012a; Park et al., 2015). Moreover, cell-penetrating and enzyme-stabilizing effects of the 30Kc19 protein have been demonstrated (Park et al., 2012b; Park et al., 2012c; Ryu et al., 2016a; Ryu et al., 2016b; Wang et al., 2011). In fact, the property of cell permeability is a very rare feature and only few cell-permeable proteins are currently revealed; Tat protein derived from human immunodeficiency virus-1 (HIV-1) (Mann and Frankel, 1991), VP22 protein derived from herpes simplex virus type 1 (HSV-1) (Brewis et al., 2000; Elliott and O'Hare, 1997), and antennapedia protein (Antp) derived from *Drosophila* (Derossi et al., 1996; Schwarze et al., 2000). Recently, artificially synthesized peptides with positive charges have also been found to be cell-permeable (Laus et al., 2000). The structural similarity of these cell-permeable proteins is that they have a protein-transducing domain (PTD) sequence that is relatively abundant in positively charged amino acids such as arginine and lysine (Gupta and Torchilin, 2006; Herce and Garcia, 2007). Utilizing these properties, proteins (Schwarze et al., 1999), nucleic acids (Abes et al., 2007), or nanoparticles (Rao et al., 2008; Torchilin, 2008) can be transmitted to the inside of the cells (Noguchi and Matsumoto, 2006; Rapoport and Lorberboum-Galski, 2009). The cell-penetrating mechanism of 30Kc19 was reported as the formation of dimers; dimerized 30Kc19 proteins enter the cells by macropinocytosis or caveolin-mediated endocytosis (Park et al., 2014). And, 30Kc19-HSA protein nanoparticles have also been used for intracellular delivery (Lee et al., 2014; Lee et al., 2016; Park et al., 2017; Hong et al., 2020).

**FIGURE 1:**
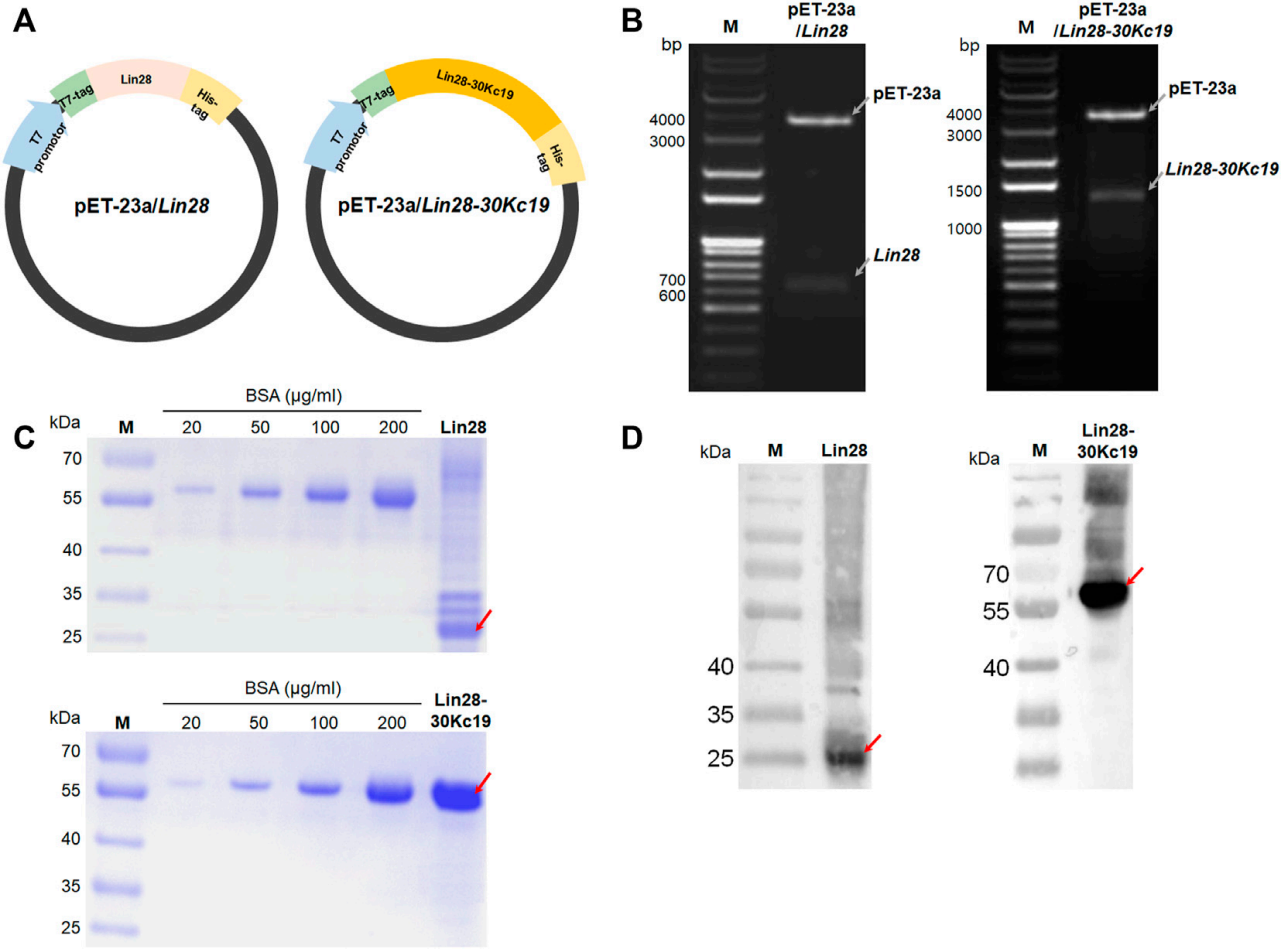
Gene construction and protein expression of Lin28 and Lin28-30Kc19. **(A)** Construction of *E. coli* expression vectors pET-23a/*Lin28* and pET-23a/*Lin28-30Kc19*. **(B)** PCR gel for confirming gene cloning of both genes, *Lin28* and *Lin28-30Kc19* in the expression vector pET-23a. Gray arrows indicate each gene of *pET-23a*, *Lin28,* and *Lin28-30Kc19*, respectively. **(C)** SDS-PAGE gel for the quantification of purified proteins, Lin28 and Lin28-30Kc19. **(D)** Western blot analysis of the recombinant proteins. Red arrows indicate the Lin28 protein and the Lin28-30Kc19 protein, respectively. M indicates the protein marker (kDa).

The objective of this study is to utilize the cell-penetrating property of the 30Kc19 protein, as a novel fusion partner of reprogramming factor Lin28 to improve the efficiency of hiPSC generation. The fusion protein was designed as follows; the cell-penetrating 30Kc19 was connected to the C-terminal domain of transcriptional factor Lin28 (Figure 1A). This resulted in the mass production of soluble recombinant Lin28-30Kc19 protein, which showed multifunctional properties; cell-penetrating by 30Kc19 and enhancing the reprogramming of the cells by transcription factor Lin28. This protein-based method is anticipated to enhance the efficiency of iPSC generation through the addition of reprogramming factors in the form of proteins, as a safer approach for future applications in regenerative medicine and *in vitro* disease modeling.

## Materials and methods

### Plasmid construction

Primers with recognition sequences for each restriction enzyme were designed in order to insert the Lin28 and 30Kc19 genes into the pET-23a expression vector (Novagen, United States). The primers for Lin28 contained *Bam*HⅠ and *Eco*RⅠ on the N and C terminals, respectively. And the primers for 30Kc19-contained *Eco*RⅠ and *Xho*Ⅰ on the N and C terminals, respectively. After amplification through a polymerase chain reaction (PCR), the PCR products were delivered into pET-23a expression vector to produce pET-23a/*Lin28* and pET-23a/*30Kc19* constructs.

### Protein expression and purification

pET-23a/Lin28 and pET-23a/Lin28-30Kc19 were transformed into *E. coli* (*Escherichia coli*), Rosetta-gami^™^ B strain (Novagen). The transformed *E. coli* were cultured in a Luria-Bertani (LB) medium (Miler, United States) containing 50 μg ml^−1^ ampicillin (Sigma-Aldrich, United States) at 37°C overnight with shaking. When OD_600_ reached 0.5, 1 mM of isopropyl-α-D-thiogalactopyranoside (IPTG; Calbiochem, United States) was added to induce protein production. *E. coli* were harvested after further incubation at 37°C for 4 h, following disruption by ultrasonication. The supernatants were filtered, and then His-tag affinity chromatography using a His-Trap HP column (GE Healthcare, Sweden) was conducted by fast protein liquid chromatography (FPLC; GE Healthcare). The binding, washing (20 mM Tris-HCl, 0.5 M NaCl, 50 mM imidazole, pH 8.0), and elution (20 mM Tris-HCl, 0.5 M NaCl, 350 mM imidazole, pH 8.0) buffers were used for protein purification. Then, a dialysis was conducted with the Tris-HCl buffer (pH 8.0) using a desalting column (GE Healthcare). The purified proteins were stored at −70°C until further use. Directly before usage, the proteins were transferred to 4°C for slow thawing.

### SDS-PAGE and immunoblotting

To confirm the size and quantify the amounts of purified proteins, a sodium dodecyl sulfate polyacrylamide gel electrophoresis (SDS-PAGE) was performed. The purified proteins were mixed with a reducing sample buffer containing SDS and β-mercaptoethanol (pH 6.8), prior to loading on a 10% SDS-PAGE gel. Through the electrophoresis, each sample was separated depending upon size. Then, the polyacrylamide gel was immersed in a Coomassie Blue staining solution for visualization. Micro bicinchoninic acid assay (BCA) kit (Thermo Fisher Scientific, United States) was used to quantify the purified proteins. To identify the purified proteins, a Western blot analysis was conducted. After the transfer of purified products on SDS-PAGE gel to a polyvinylidene difluoride (PVDF) membrane, proteins were labeled with an anti-T7-tag antibody (Abcam, United Kingdom) and an anti-rabbit HRP-conjugated antibody (Millipore, United States). Lumina Forte Western HRP substrate (Millipore) was used to visualize proteins on the G-Box Chemi XL system (Syngene, United Kingdom).

### Cultivation of HDF

Human dermal fibroblasts (HDFs; ATCC® PCS-201-010, United States) were maintained by Dulbecco’s Modified Eagle’s Medium (DMEM; HyClone, Logan, UT, United States) supplemented with 10% fetal bovine serum (Gibco, Waltham, MA, United States), 100 U/ml penicillin, and 100 μg/ml streptomycin (Gibco) in 5% CO_2_ humidified incubator at 37°C. When the confluency reached about 70%, HDFs were transferred in a 1:5 ratio. HDFs used in this study were under passage 10 in all experimental steps.

### Cell-penetration assay

After 1 × 10^5^ cells of HDFs were seeded onto a well of a six-well plate and incubated at 37°C overnight, proteins at concentrations of 10 and 20 μg ml^−1^ were treated to the HDFs, and then incubated at 37°C for another 24 h, respectively. In order to visualize cellular uptake of the Lin28-30Kc19 protein, immunocytochemical analysis was performed. The cells were fixed with 4% paraformaldehyde (Sigma-Aldrich) for 15 min and then permeabilized with 0.05% Tween-20 (Amresco, United States) for 2 h. An anti-T7-tag rabbit antibody (Abcam) and secondary Alexa-488 labeled anti-rabbit goat antibody (Abcam) were utilized. After washing with PBS several times, Hoechst 33342 (Life technologies, United States) was treated to stain nuclei for 10 min. Then, a confocal laser microscope (Olympus, United States) was used to visualize the fluorescence. Western blotting was also used, as mentioned previously, to detect the cellular uptake of the proteins.

### Cytotoxicity assay

For the cytotoxicity analysis of the Lin28-30Kc19 protein to HDFs, 5 × 10^3^ cells were seeded onto a 96-well plate. The cells were then cultured at 37°C for 24 h, until the cell confluency reached about 70%. And the protein was treated to the cells at concentrations of 0–50 μg ml^−1^, followed by incubation at 37°C for another 24 h. Then, the cell counting kit-8 (Dojindo, Korea) was used according to the manufacturer’s method. Absorbance was measured at 450 nm using an ELISA reader. All values are presented as mean ± standard deviation (s.d.) and all of the experiments were performed thrice, resulting in comparison with the control using *t*-test.

### Retrovirus production

GP2-293 packaging cells were cultured, and 1 × 10^6^ cells were seeded onto a 100 mm dish followed by overnight cultivation at 37°C. The cells were transfected with Oct4 (Addgene plasmid #17217, United States), Sox2 (Addgene plasmid #17218), c-Myc (Addgene plasmid #17220), and Klf4 (Addgene plasmid #17219) using Lipofectamine® 3000 (Invitrogen, United States) according to the manufacturer’s instructions. As an envelope vector, VSV-G (Addgene plasmid #14888) was used, and as a reference gene, DsRed (Addgene plasmid #22724) was prepared. After the treatment of each transcription factor with an envelope vector or DsRed, GP2-293 cells were cultured at 37°C for 48 h. Then, the virus-containing medium was collected and filtered through 0.45 μm membrane filters (Pall Corporation, United States), and the viruses were precipitated using Retro-Concentin^™^ (System Biosciences, United States). The precipitated viruses were suspended using DMEM and stored at −70°C for further use.

### Generation of hiPSCs

For the reprogramming of HDFs, cells were prepared at 1 × 10^5^ cells per 35 mm dish. Cell culture medium was changed to fresh medium, containing four transcriptional factors in the form of viruses, supplemented with 8 μg ml^−1^ of polybrene (Sigma-Aldrich) for transduction. After the cells were incubated with viruses at 37°C for 24 h, they were transferred to a six-well plate at 1 × 10^5^ cells per a well. The next day, the medium was replaced with a fresh medium. And from day 4, the medium was switched to an Essential 8^™^ medium (Gibco, United States) and the medium was replaced every day. The Lin28-30Kc19 protein was treated with an Essential 8^™^ medium from the fourth day, and the treatment interval and concentration of the proteins varied as results. In brief, in the experimental group with a 24 h interval, the recombinant protein was treated everyday from day 4 to day 13. Therefore, the Lin28-30Kc19 protein was treated to HDFs 10 times for 14 days. In the experimental group with 48 h interval, the protein was treated every other day from day 4 to day 6 (for characterization of hiPSCs on early stage) or to day 12. So, the recombinant protein Lin28-30Kc19 was treated to HDFs 2 times (on day 4 and 6) in the hiPSCs analyzed on day 7, whereas the protein was treated to the cells five times (on day 4, 6, 8, 10, and 12) in the iPSCs prepared on day 14.

### Alkaline phosphatase staining

To detect pluripotency of the generated hiPSCs, alkaline phosphatase (AP) staining was used. Cells were washed twice with PBS after fixation and then stained using a leukocyte alkaline phosphatase kit (Sigma-Aldrich). The stained cells were observed with an optical microscope (Olympus).

### Statistical analysis

All the experiments were conducted at least thrice. Data are expressed as the mean ± SD. Statistical significance was determined using the Student’s t-test to compare two groups through the SigmaPlot. For all experiments, statistical significance was represented by * for *p* < 0.05, ** for *p* < 0.01, and *** for *p* < 0.001.

## Results

### Expression and purification of Lin28-30Kc19 protein

To construct vectors of pET-23a/*Lin28* and pET-23a/*Lin28-30Kc19* for the protein expression, two types of genes were prepared; *Lin28* and *30Kc19* (Figure 1A). For the Lin28 gene, obtained from pSin-EF2-LIN28-Pur (Addgene plasmid #16580), primers contained *Bam*HⅠ and *Eco*RⅠ on the N and C terminals, respectively. The 30Kc19 gene was obtained from the vector pET-23a/*GFP-30Kc19* used in the previous research. The primers for 30Kc19 include *Eco*RⅠ on the N terminal and *Xho*Ⅰ on the C terminal. After amplifying through PCR, those two genes were inserted into the pET-23a expression vector. Therefore, in order to confirm the cloning of pET-23a/*Lin28* and pET-23a/*Lin28-30Kc19*, suitable restriction enzymes were used as follows; *Bam*HⅠ and *Eco*RⅠ for pET-23a/*Lin28*, and *Bam*HⅠ and *Xho*Ⅰ for pET-23a/*Lin28-30Kc19*. As pointed with gray arrows in Figure 1B, the gene of the backbone plasmid pET-23a was observed at 3,666 bp in both pET-23a/*Lin28* and pET-23a/*Lin28-30Kc19*. And the inserted *Lin28* and *Lin28-30Kc19* appeared at 630 bp and 1374 bp, respectively. The recombinant proteins, produced by transforming the cloned pET-23a/*Lin28* and pET-23a/*Lin28-30Kc19* plasmids into *E. coli*, have a T7-tag at the N terminal and 6-Histidine-tag at the C terminal.

Proteins purified using FPLC were isolated by 10% SDS-PAGE and their sizes were confirmed by Coomassie brilliant blue staining (Figure 1C) and Western blot (Figure 1D). As described in the image, vague bands of the Lin28 protein were observed at the position of 29 kDa and the obvious Lin28-30Kc19 protein had bands at the position of 60 kDa, resulting in the same sizes of the expected protein sizes, respectively. For quantification of purified proteins, a micro BCA kit was used. Also, the thickness and density of the bands was compared with that of bovine serum albumin (BSA; Millipore, United States) band for a complemental analysis. Consequently, by measuring the SDS-PAGE gel images through the ImageJ software, the purity and final concentration after purification, as well as the yield of the Lin28 protein and the Lin28-30Kc19 protein were calculated. Protein purities were 27.2 and 96.2% for Lin28 and Lin28-30Kc19, respectively. In terms of the final concentration after purification, the Lin28 protein was 0.131 mg ml^−1^, and the Lin28-30Kc19 protein was 0.742 mg ml^−1^. The yield of the Lin28 protein was 0.328 mg, when 1 L of *E. coli* cells was cultured and the value of Lin28-30Kc19 protein was 3.71 mg L^−1^.

### *In vitro* properties of the Lin28-30Kc19 protein

**FIGURE 2:**
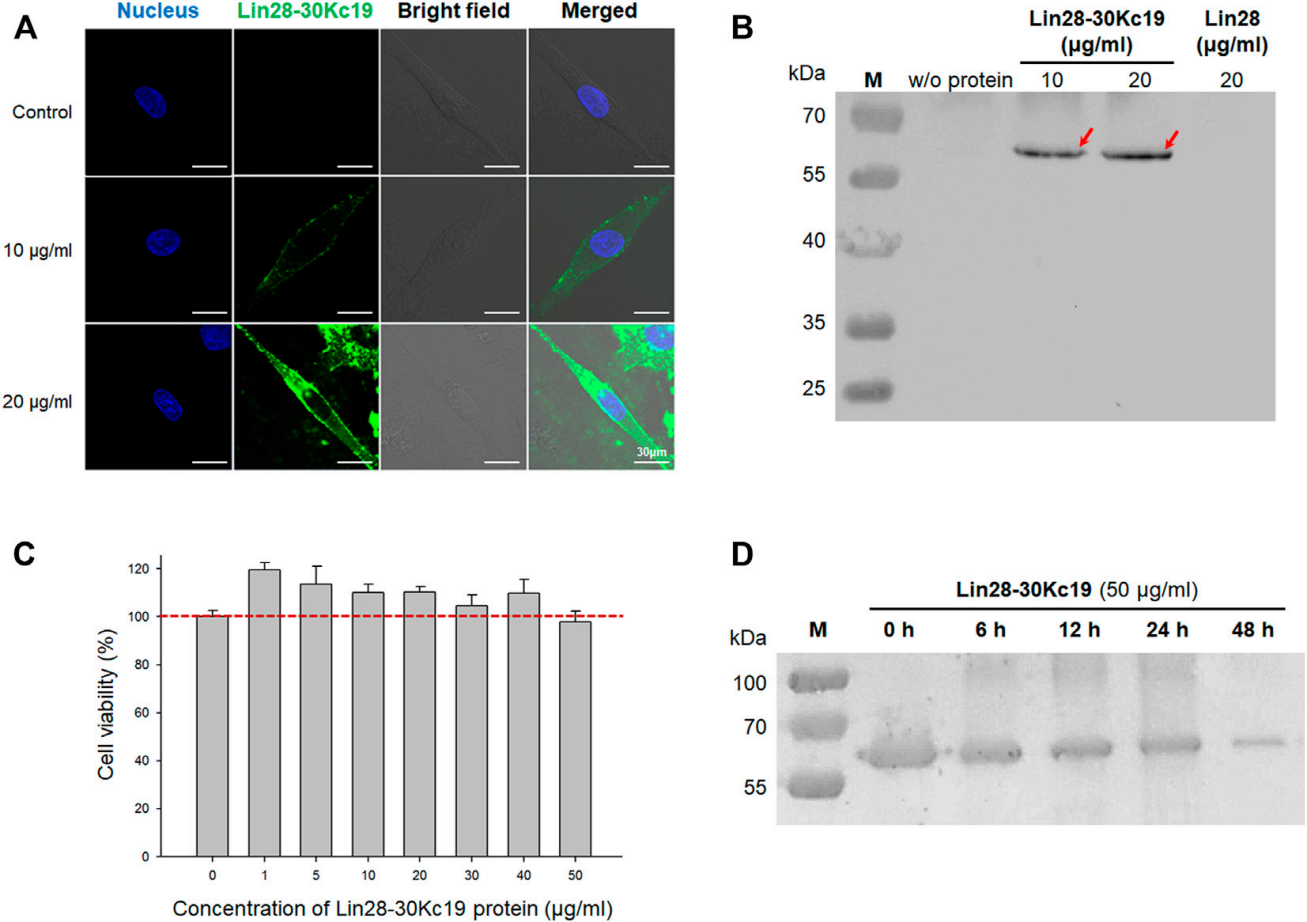
*In vitro* properties of the Lin28-30Kc19 protein. **(A)** Confocal images for detecting the intracellular delivery of the Lin29-30Kc19 protein. HDFs treated with 10 and 20 μg ml^−1^ of the Lin28-30Kc19 protein were observed, respectively. Blue fluorescence indicates the nuclei and green fluorescence indicates the Lin28-30Kc19 protein. Scale bars indicate 30 μm. **(B)** Western blot analysis of the cytosolic fraction of protein-treated cells to confirm the cell penetration property. After cultivation of cells with proteins for 24 h, cells were disrupted and then cell lysates were analyzed. Red arrows indicate the intracellularly delivered Lin28-30Kc19 protein. **(C)** Cytotoxicity of the Lin28-30Kc19 protein, depending on the concentration of the protein. HDFs were treated with each concentration of the Lin28-30Kc19 protein for 24 h and then cell viability was measured. The red dotted line indicates 100%, which is the value of the control group (untreated cells). *n* = 3. **(D)** *In vitro* stability of the Lin28-30Kc19 protein by Western blotting. HDFs treated with 50 μg ml^−1^ of the Lin28-30Kc19 protein were analyzed after 0, 6, 12, 24, and 48 h, respectively.

To detect the *in vitro* properties of the Lin28-30Kc19 protein, cell-penetrating activity, cytotoxicity, and *in vitro* stability were analyzed. Cell permeability of the fusion protein, Lin28-30Kc19, was analyzed by immunocytochemistry and Western blot (Figures 2A,B). According to Figure 2A, the Lin28-30Kc19 proteins were treated to HDFs for 24 h, and then the protein was detected by green fluorescence. As shown in the immunocytochemical images, the Lin28-30Kc19 protein was intracellularly delivered, regardless of the concentration. In both of the cells treated with 10 and 20 μg ml^−1^ of the Lin28-30Kc19 protein each, green fluorescence was detected in cytosols, whereas in control (non-treated cells) there was no green fluorescence in the cells. Compared with cells treated with 10 μg ml^−1^ of the Lin28-30Kc19 protein, cells with 20 μg ml^−1^ of Lin28-30Kc19 showed enhanced fluorescence. Also, the HDFs cultured with the Lin28-30Kc19 protein (10 and 20 μg ml^−1^, respectively) and the Lin28 protein for 24 h were analyzed through immunoblotting (Figure 2B). After the collection of cell lysates, they were separated by 10% SDS-PAGE for Western blotting. According to the results, the band detecting intracellularly delivered proteins was not observed in cells with Lin28, such as the control group (non-treated cells). Whereas, the cells treated with the Lin28-30Kc19 protein showed an obvious band at the expected position of size, regardless of the concentration of the protein. Therefore, the Lin28 protein which does not have cell permeability could penetrate cells when combined with 30Kc19, in the form of the Lin28-30Kc19 fusion protein.

Due to their cell penetration activity, the cytotoxicity of the Lin28-30Kc19 proteins was analyzed (Figure 2C). The HDFs with various concentrations (0–50 μg ml^−1^) of the fusion proteins were investigated after 24 h of incubation. According to the results, the viability of cells treated with proteins was increased (1–40 μg ml^−1^), when compared with the control group (non-treated cells; 100%). In particular, the lower the concentration of the treated Lin28-30Kc19 protein, the greater the increase in cell viability. Although the cell viability was lower than 100% in HDFs treated with 50 μg ml^−1^ of the recombinant protein, it was confirmed that cell-penetrating Lin28-30Kc19 protein does not have toxicity in cells.

In Figure 2D, denaturation of the Lin28-30Kc19 protein was investigated in order to confirm *in vitro* stability of the protein and to determine a practical interval for protein treatment to the cells. 50 μg ml^−1^ of the fusion protein was incubated and then collected after 6, 12, 24, and 48 h, respectively. According to the Western blotting result, the band intensity for the Lin28-30Kc19 protein gradually decreased as a function of time, compared to the control group (0 h). The largest decrease in the amount of remained proteins was observed between 24 and 48 h.

### Optimization of the protein treatment for reprogramming fibroblasts to hiPSCs

**FIGURE 3:**
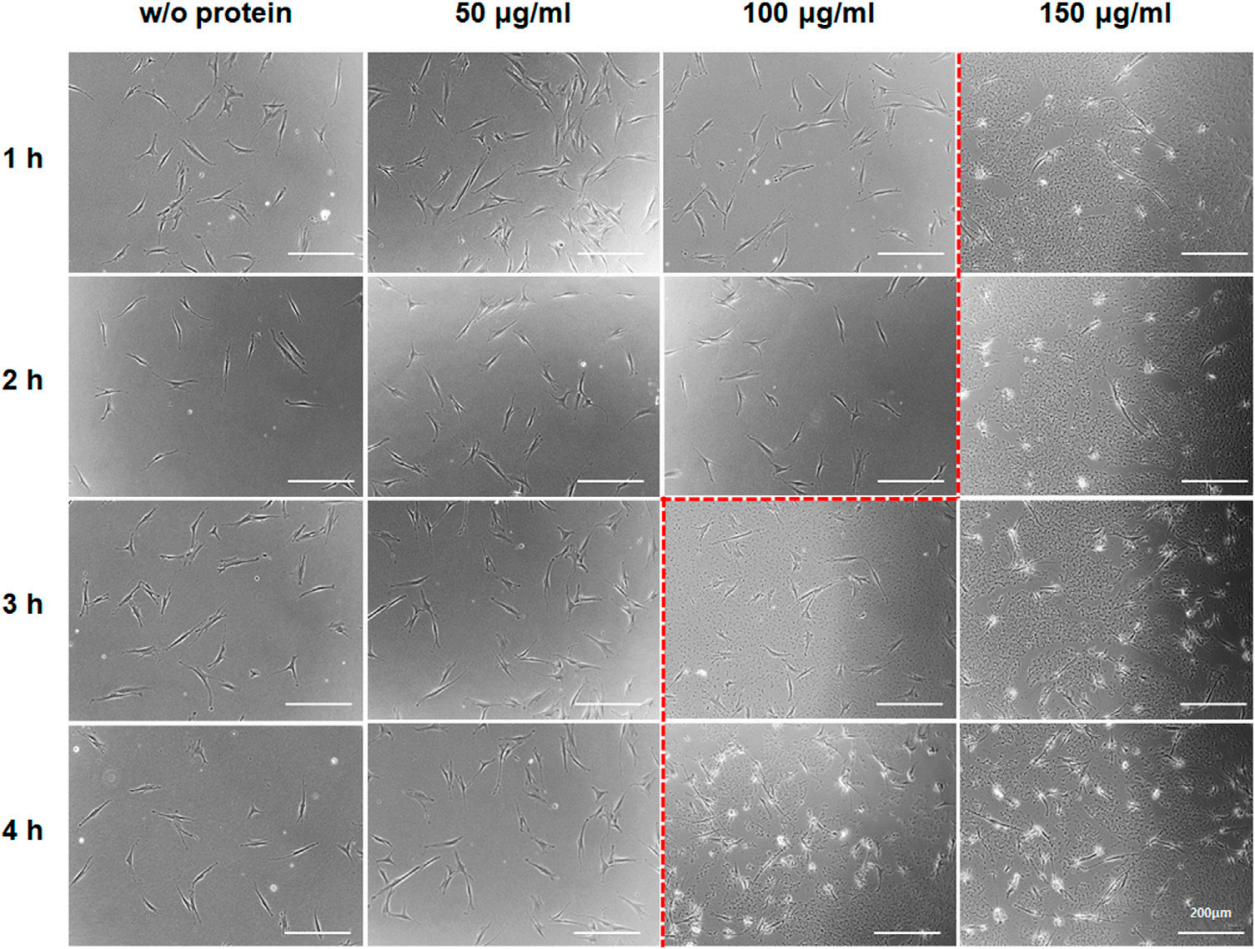
Aggregate-like forms of the Lin28-30Kc19 protein. HDFs treated with the Lin28-30Kc19 protein were observed through a microscope, depending upon the concentration of the protein and incubation time with the protein. Based on the red dotted line, the right region showed an aggregation tendency of the protein. Scale bars indicate 200 μm.

**FIGURE 4:**
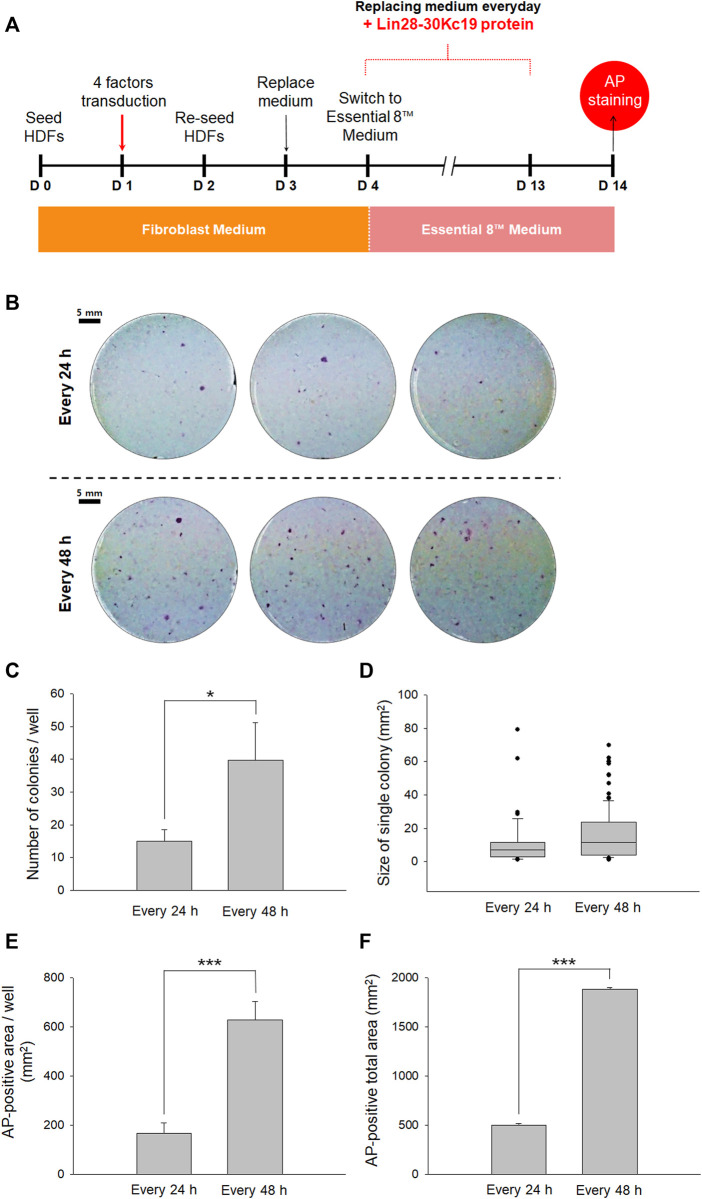
Optimal protein treatment interval for reprogramming fibroblasts to hiPSCs. **(A)** Timeline for determining the optimal interval of the protein treatment. From Day 4, the cell culture medium was switched from a fibroblast medium to an Essential 8^™^ medium, and the Lin28-30Kc19 protein was treated every 24 or 48 h, respectively. Then, the cells were analyzed by AP staining on day 14. **(B)** Naked eye images of the AP-stained cells treated with the Lin28-30Kc19 protein for every 24 or 48 h, respectively. Scale bars indicate 5 mm. **(C)** Number of AP-stained colonies in a well. The AP-positive colonies were counted and compared in 24 and 48 h groups. **(D)** Size of the AP-positive single colony. Each AP-stained area was quantified and compared in the 24 and 48 h groups. **(E)** The AP-positive area in a well. Positively detected area was measure and compared in 24 and 48 h groups. **(F)** Total area positively stained with AP in three wells. The area of AP-positive colonies was summed up and compared in 24 and 48 h groups. *p < 0.05 and ***p < 0.001. *n* = 3.

To maximize the productivity of hiPSC, an optimal condition for the protein treatment to cells was analyzed; investigation to define an appropriate protein concentration (Figure 3) and an analysis for effective protein treatment interval (Figure 4). First, in order to determine the efficient concentration of the protein, the aggregation tendency of the protein was observed (Bucciantini et al., 2002), depending upon protein concentrations and the incubation time (Figure 3). After HDFs were cultured with 50, 100, and 150 μg ml^−1^ of the Lin28-30Kc19 protein, respectively, change over time was observed using a microscope. At a concentration of 50 μg ml^−1^, there was no protein aggregation even after 4 h. However, from a concentration of 100 μg ml^−1^, the aggregate-like forms were observed from 3 h of incubation. In addition, an obvious aggregation of protein occurred less than 1 h, when 150 μg ml^−1^ of the protein was treated. Therefore, it was determined not to exceed 100 μg ml^−1^, and 50 μg ml^−1^ of Lin28-30Kc19 would be prefer for *in vitro* treatment to HDFs for hiPSC generation.

Second, to suggest an effective protein treatment interval for the generation of hiPSCs, two types of intervals were analyzed by AP staining on the 14th day (Figure 4A). For reprogramming, the HDFs were treated with four transcriptional factors in the form of viral vectors and the Lin28-30Kc19 recombinant protein was additionally treated. Cells with the Lin28-30Kc19 protein every day and cells with the recombinant protein every second day were compared in naked eyes (Figure 4B). According to Figure 4C, hiPSCs treated with the Lin28-30Kc19 protein per 48 h showed statistically increased number of colonies (over twice) compared with the hiPSCs with a 24 h-interval protein treatment. In Figure 4D, the size of a single colony was analyzed, and compared in 24 and 48 h groups. According to the results, the average value was 11.1 and 15.8, and the median value was 6.9 and 11.4 in the 24 and 48 h groups, respectively. The AP-positive area was also measured and compared in the 24 and 48 h groups. Both AP-positive area in a well (Figure 4E) and the total area in three wells (Figure 4F) significantly increased in the 48 h groups (almost four times) comparing with the 24 h groups. Therefore, 48 h was chosen as an optimal interval for the recombinant protein treatment in this study.

### Characterization of hiPSCs generated using the Lin28-30Kc19 protein

**FIGURE 5:**
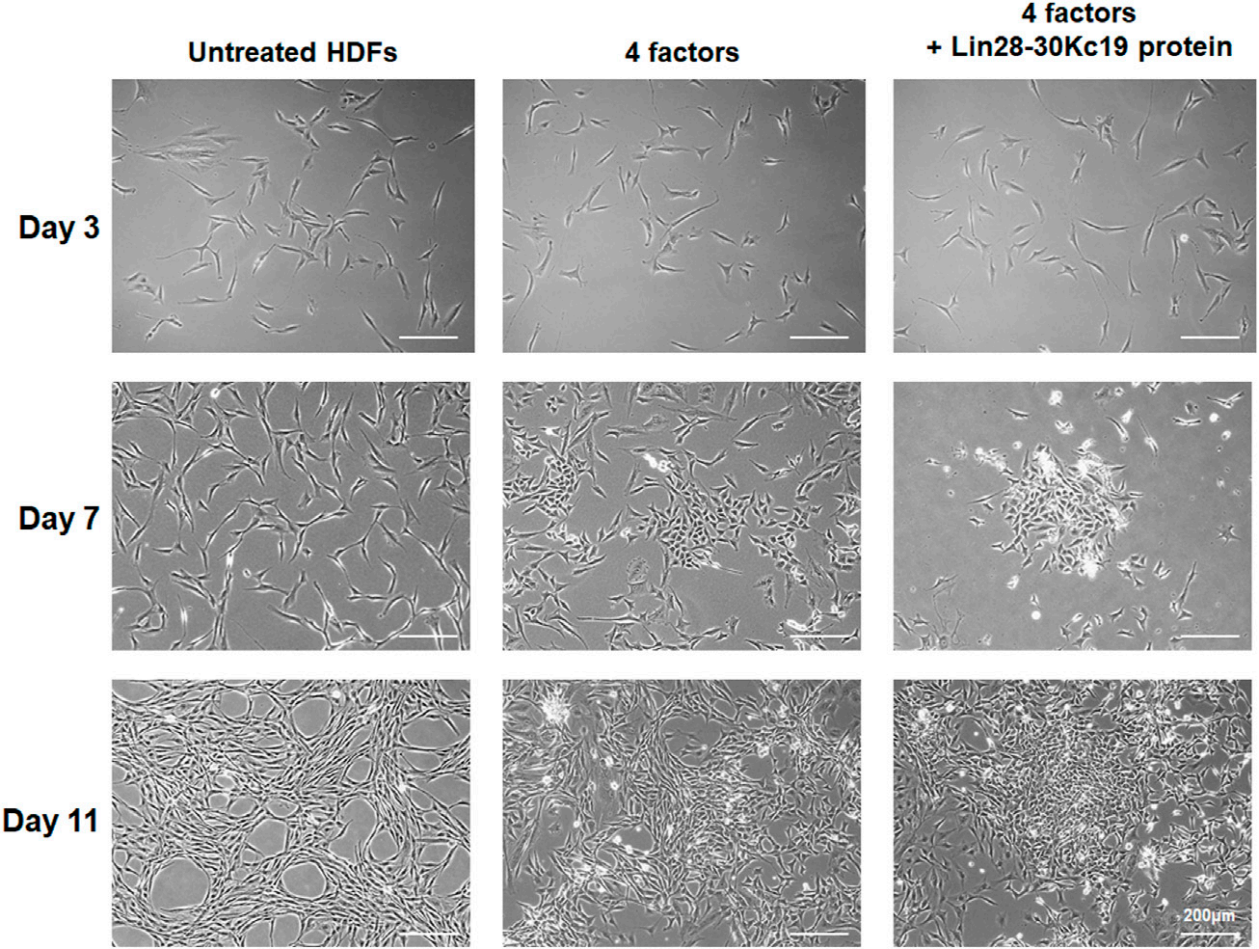
Morphological comparison of hiPSCs. Morphological changes of the fibroblasts were observed in untreated HDF (left), hiPSCs generated by four factors (middle), and hiPSCs produced by four factors with the Lin28-30Kc19 protein (right), at days 3, 7, and 11, respectively. Scale bars indicate 200 μm.

After optimizing the protein treatment condition for the generation of hiPSCs, HDFs were observed during the 14 days of reprogramming. The concentration of the Lin28-30Kc19 protein treated to cells for hiPSC generation was 50 μg ml^−1^, and the protein was treated every 48 h. In Figure 5, the morphological change was compared in untreated HDFs, HDFs treated with four factors, and the HDFs with four factors and the Lin28-30Kc19 protein. According to the microscopic images, unlike the morphological characteristics of untreated HDFs, cells treated with four factors with or without the Lin28-30Kc19 protein showed a gradual change into plump shapes, resulting in a formation of colonies. Particularly, the cells treated with both four factors and the fusion protein gathered to form larger and compact colonies over time, compared to other groups.

**FIGURE 6:**
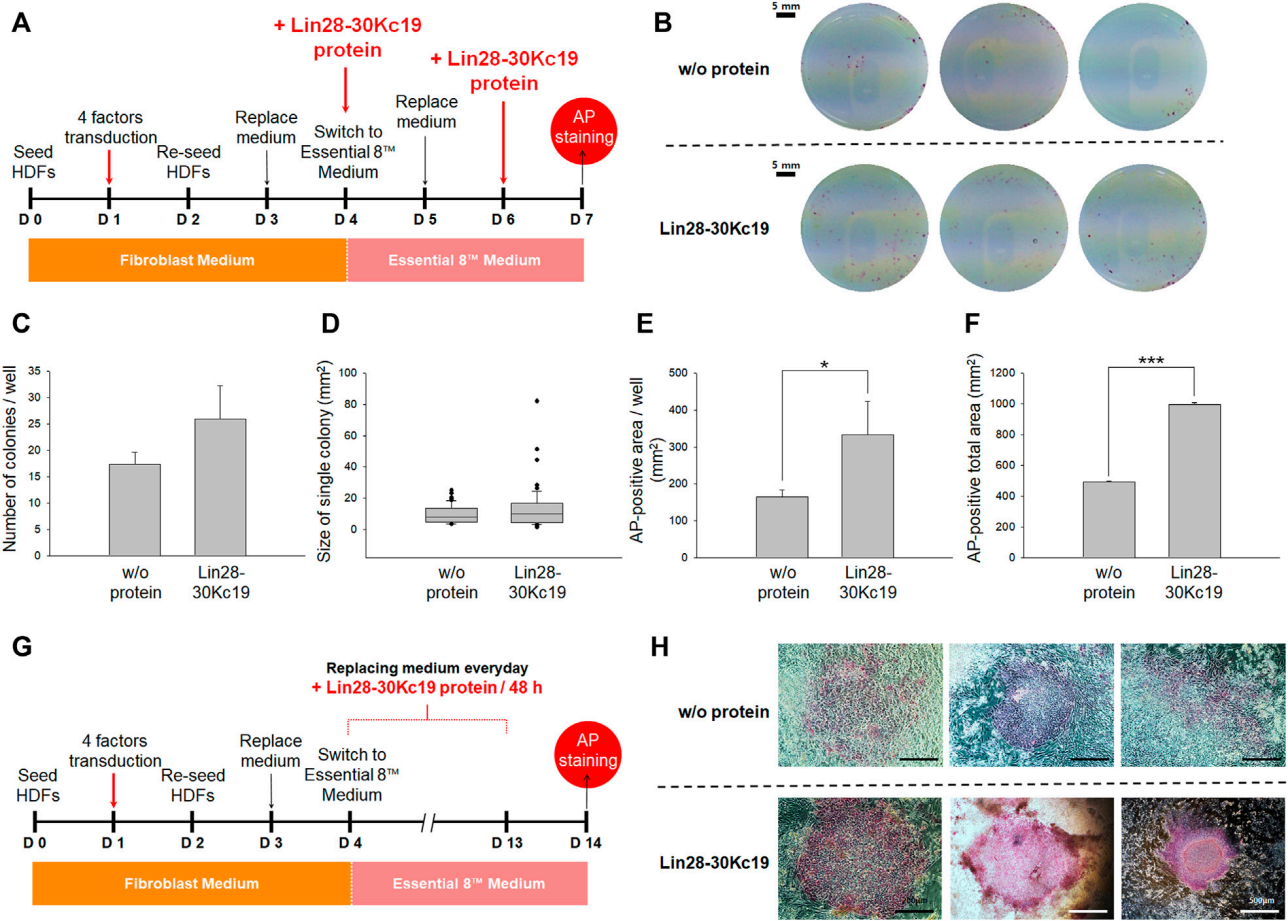
Characterization of hiPSCs generated by the addition of the Lin28-30Kc19 protein. **(A)** Timeline for characterization of hiPSCs at an early stage (day 7). **(B)** Naked eye images to detect reprogramming efficiency on day 7. HDFs treated with the Lin28-30Kc19 protein on days 4 and 6 were analyzed by AP staining. Scale bars indicate 5 mm. **(C)** Number of AP-stained colonies in a well on day 7. The AP-positive colonies were counted and compared in the without and with protein groups. **(D)** Size of an AP-positive single colony on day 7. Each AP-stained area was quantified and compared in the without and with Lin28-30Kc19 protein groups. **(E)** The AP-positive area in a well on day 7. Positively detected area was measured and compared without and with the recombinant protein groups. **(F)** The total area positively stained with AP in three wells on day 7. The area of AP-positive colonies was summed up and compared in without and with the protein groups. *p < 0.05 and ***p < 0.001. *n* = 3. **(G)** Timeline for the characterization of hiPSCs after passaging (day 14). **(H)** Microscopic images to confirm reprogramming efficiency on day 14. HDFs treated with the Lin28-30Kc19 protein on days 4, 6, 8, 10, and 12 were analyzed by AP staining. Scale bars indicate 500 μm.

To confirm hiPSC generation and its pluripotency, AP staining was conducted at days 7 and 14, respectively, comparing the two groups; HDFs treated with four factors, and HDFs with four factors and the Lin28-30Kc19 protein (Figure 6). As described in Figure 6A, reprogramming efficiency in an early stage was determined by AP staining on day 7. Cells added with the Lin28-30Kc19 protein showed more AP-stained colonies, when observed by naked eyes, compared with HDFs reprogrammed by only four factors without protein (Figure 6B). According to Figure 6C, hiPSCs treated with the Lin28-30Kc19 protein showed an increased number of colonies (about 1.5 times) compared with the hiPSCs without protein. In Figure 6D, the size of a single colony was analyzed, and compared in cells with or without protein. According to the results, the average values were 9.4 and 12.7, and the median values were 8.1 and 10.0 in without and with groups, respectively. The AP-positive area was also measured and compared without protein and with the Lin28-30Kc19 groups. Both AP-positive area in a well (Figure 6E) and the total area in the three wells (Figure 6F) significantly increased in cells treated with the recombinant protein Lin28-30Kc19 (about twice) compared to the without protein groups. Likewise, cells observed on day 14 showed a similar tendency (Figures 6G, H). In hiPSCs generated by four factors and the Lin28-30Kc19 protein, AP-stained colonies showed clearer red color, and the size of the colonies was larger, when compared with the hiPSCs produced by only four factors.

## Discussion

Like ESCs, the iPSCs have unique characteristics such as the self-renewal property and pluripotency. Since iPSCs are free from ethical issues, which have been raised for ESCs due to the origination of the cells, the use of iPSCs has overcome the limitations in using ESCs. Thus, the iPSCs have been regarded as one of the most promising techniques for the therapeutic application of stem cells. However, the efficiency of iPSC generation needs further improvement. According to recent studies, in addition to the four factors (Oct4, Sox2, c-Myc, and Klf4) (Nakagawa et al., 2008), other additional transcriptional factors have been complemented. Lin28 has been known to increase the efficiency of iPSC generation, when co-utilized with the four factors (Yu et al., 2007). In 2009, according to Kim et al. and Zhou et al., the mouse and human iPSCs were generated using four reprogramming factors in the form of protein, binding to a cell-permeable protein. However, the efficiency of iPSC generation was not sufficient, and thus multiple transfections were needed. Moreover, it was challenging to produce soluble recombinant proteins to use for iPSC generation. Though there have been many efforts to improve the production of transcription factors in the form of proteins, the inclusion body forming–reprogramming factors in the microbial expression system and low production in the mammalian system are still remained limitations (Pan et al., 2015). Therefore, improving the efficiency of iPSC generation has become an important issue, and many attempts have been made to develop methods for generating the iPSCs.

**FIGURE 7:**
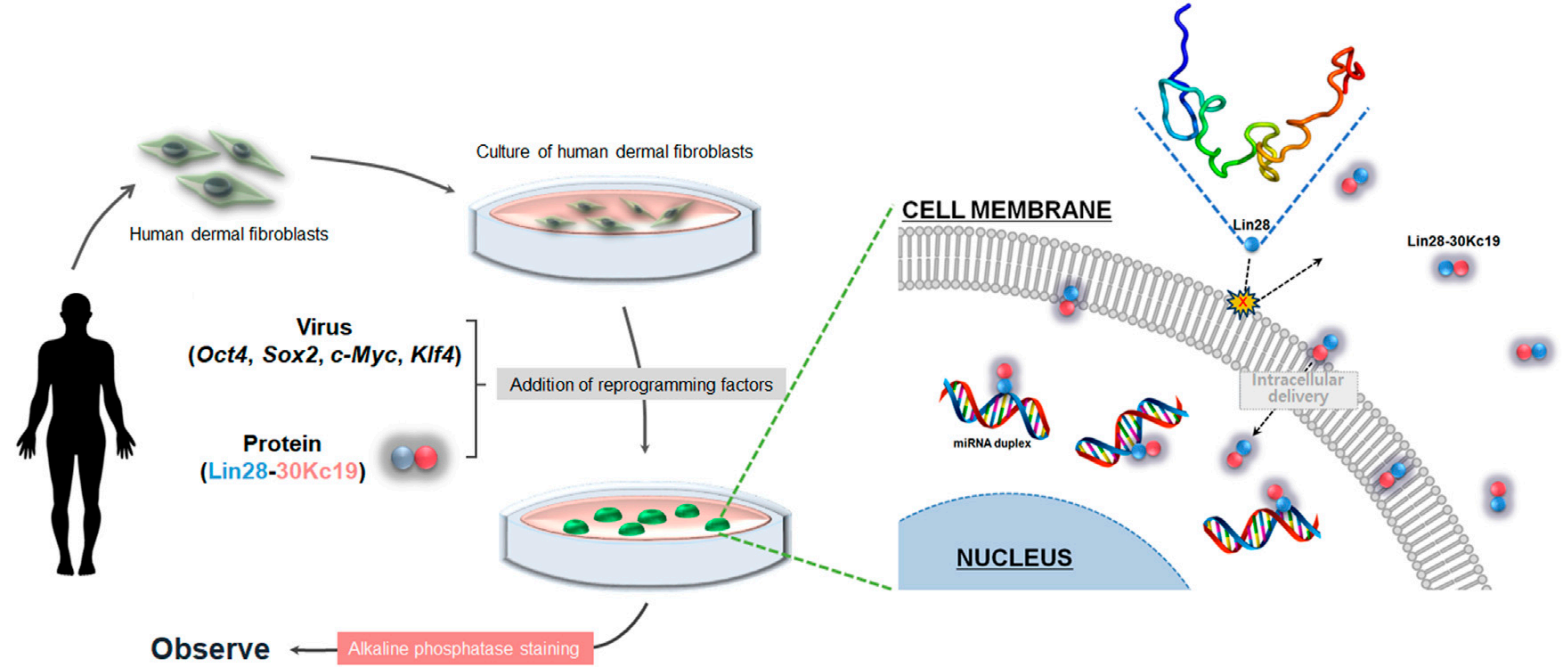
Overall schematic of hiPSC generation using the Lin28-30Kc19 protein. Addition of the Lin28-30Kc19 protein improved the efficiency of hiPSC generation, due to the cell-penetrating and protein-stabilizing properties of the 30Kc19 protein, enabling intracellular delivery and maintenance of the stability of transcription factor, Lin28.

In this study, we tried to improve the hiPSC productivity by adding Lin28, which is one of the reprogramming factors and is also known as a significant enhancer for hiPSC generation (Figure 7). In order to efficiently deliver the Lin28 protein into cells and protect important properties of the protein, we suggested in the form of a fusion protein, Lin28-30Kc19. According to a former study related to the production of the Lin28 recombinant protein (Pan et al., 2015), the Lin28 was produced in the form of a TAT-fused protein. But the recombinant protein was not produced in a secreted form under the insect cell expression system. This is supported by the highly insoluble property of reprogramming factors when produced in the *E. coli* expression system (Zhou et al., 2009). Due to the inclusion body-forming property, the purification of the recombinant form of reprogramming proteins is a labor-intensive and costly process, making its practical use difficult. Indeed, although we tried to express the Lin28 in a soluble form, it was not possible (Figure 1). Therefore, in this study, to improve the soluble expression of Lin28 and enable the mass production of the protein, we fused Lin28 with 30Kc19 in the form of recombinant protein Lin28-30Kc19 (Ryu et al., 2016b). Through the fusion with the 30Kc19 protein, the solubility of the Lin28 protein was increased by about five times when produced in the form of a recombinant protein, Lin28-30Kc19, when compared to the Lin28 protein alone (Figure 1C). Moreover, intracellular delivery across the cell membrane was also observed for the Lin28-30Kc19 protein based on the former studies (Ryu et al., 2016a; Park et al., 2017) in Figures 2A,B. Regardless of the concentrations, the recombinant protein Lin28-30Kc19 showed a cell-permeable property by not only the immunocytochemical analysis but also immunoblotting. According to the fluorescence results, the fusion protein Lin28-30Kc19 was observed in the cytosol and even in nuclei. And the sole Lin28 protein, which could not be produced soluble enough, it did not show intracellular delivery in the Western blotting results. Through a cytotoxicity test of the fusion protein, the relative viability of the cells was compared depending upon the concentration of the treated Lin28-30Kc19 protein. There was a slight increase in cell viability in cells treated with the fusion protein, compared to control (untreated cells) (Figure 2C). Therefore, we concluded that the recombinant protein Lin28-30Kc19 does not present cytotoxicity on HDFs. Based on the studies regarding the effect of Lin28 on cells, it is presumed that the increase of the cell viability with the Lin28-30Kc19 recombinant protein would be associated with the property of the Lin28 protein, which regulates intracellular glucose metabolism (Zhu et al., 2011; Zhang et al., 2012). Moreover, Lin28 is known to be involved in cell proliferation in several cell types possessing the potential of differentiation such as stem cells (Shyh-Chang and Daley, 2013; Shyh-Chang et al., 2013). However, according to those related studies, the cell-growing effect of Lin28 has been reported only in cells with a differentiable property. Thus, the cell viability and cellular growth should be distinguished avoiding confusions in this study, in which only the differentiated somatic cells (HDFs) were used for experiments. The cell counting kit-8 (CCK-8) in this study was utilized for the detection of viable cells. According to the mechanisms of the CCK-8, water-soluble tetrazolium salt was reduced by dehydrogenase activities in live cells, resulting in the generation of an orange color formazan dye, which is detectable (Yang et al., 2021). Therefore, CCK-8 can be originally used for the cytotoxicity test; however, it does not directly connect to cell proliferation. According to the results, the increase of cell viability was prominent in low concentrations of the protein (1–10 μg ml^−1^); the lower the concentration of the Lin28-30Kc19 protein, the greater the increase of cell viability is. It is considered to be due to the tendency to form protein aggregation at high concentrations (Figure 3). From the protein stability analysis, the stable form of the recombinant protein gradually decreased over time. Especially, the greatest reduction was between 24 and 48 h (Figure 2D). Due to technical limitations, the expression of soluble Lin28 was not possible, and thus the increment of protein stability caused by the 30Kc19 protein could not be compared.

Although we confirmed that 50 μg/ml of the Lin28-30Kc19 protein did not show cytotoxicity, a slight decrease in the cell viability was observed at 50 μg/ml concentration of the Lin28-30Kc19 protein (Figure 2C). So, the suitability of the recombinant protein at 50 μg/ml concentration needs to be verified by an additional experiment. Therefore, in order to provide proper evidence, cellular morphology was observed when incubated with different concentrations (50, 100, and 150 μg/ml, respectively) of the recombinant Lin28-30Kc19 protein in Figure 3. According to the previous study (Bucciantini et al., 2002), the cytotoxicity of the protein has been detected and analyzed by morphological observation of the cells and the form of protein. When an aggregation of protein was observed in microscopic images, it was considered to be toxic to cells. Therefore, we conducted microscopic observations of the cells incubated with recombinant protein Lin28-30Kc19, in order to insist the cytotoxicity of the protein over 100 μg/ml and to exclude the toxic conditions (100 and 150 μg/ml). When we observed the aggregate-like forms (Amen and Kaganovich, 2015; Alberti et al., 2017) of the Lin28-30Kc19 protein, there was obvious protein aggregations at a concentration of 100 μg ml^−1^ after 3 h, and 150 μg ml^−1^ less than only 1 h (Figure 3). Therefore, the optimal and stable concentration of protein was defined as 50 μg ml^−1^. Also, in order to determine the protein treatment condition for the reprogramming of HDFs into hiPSCs, an assay regarding the interval of protein treatment was performed. According to Figures 4B–F, 48 h was decided as optimal. Cells treated with the Lin28-30Kc19 protein bidaily showed an enhanced hiPSC generation, comparing with cells treated daily with the protein. This difference in efficiency is considered to be due to the protein aggregate-like forms caused by accumulation that happens when proteins were treated frequently, even though the concentration was not too high. We observed cell detachment because of protein aggregations, in the cells treated with the protein every 24 h.

The Lin28 could not be expressed in a soluble form due to technical limitations. Therefore, in Figures 5, 6, the comparison of hiPSC generation efficiency between the Lin28-30Kc19 recombinant protein and the sole Lin28 protein was not possible.

According to the results of hiPSC confirmation by AP staining in the early stage of culture (day 7), HDFs treated with the four factors and the Lin28-30Kc19 protein showed an increased number of colonies with evident red, compared with control which was reprogrammed by only four factors (Figure 6). It indicated the excellent effect of Lin28-30Kc19, which greatly improved the efficiency of reprogramming, even if the protein was treated only twice. According to the analysis performed on day 14, similar results were observed where the size of individual colonies more than doubled, the degree of color in AP-stained colonies was stronger, and the staining boundaries were clear when treated with both the four factors and the Lin28-30Kc19 protein (Figure 6H).

As a result, the effect of the Lin28-30Kc19 protein on hiPSC generation could be validated by comparing cells analyzed on day 7, in which the protein was treated on day 4 and 6 (Figure 6), and the cells investigated on day 14, in which the protein was treated on days 4, 6, 8, 10, and 12 (Figure 4). The major differences of the two groups in the experimental condition were as follows: First, the periods of incubation for reprogramming were 7 and 14 days, respectively. Second, the recombinant protein was treated to the cells twice and 5 times, respectively. The average number of AP-stained colonies was 26.0 in cells on day 7 with protein treatment twice. And the value was 39.6 in the cells on day 14 with protein treatment 5 times. During the next 7 days, the number of colonies with pluripotency increased about 1.5 times. Regarding the size of a single colony, the average value was 12.8 and 15.8 mm^2^ in cells on days 7 and 14, respectively, indicating that there was no significant difference in the size of a single colony caused by longer cultivation (7 days) and more protein treatment number (three times). However, the summed value of the AP-positive area increased in the cells on day 14, due to the number of colonies. In this regard, it is considered that the recombinant protein Lin28-30Kc19 contributes in reprogramming the HDFs into hiPSCs through the generation of iPSC colonies, increasing the number of pluripotent colonies, rather than enlarging the size of the colonies.

Therefore, it was found that the Lin28-30Kc19 protein has an effect of increasing the number and size of the AP-positive hiPSC colonies rather than the conventional method, using only the four factors. To improve and deepen this study, additional characterization of the generated hiPSCs is needed more than AP staining. Then, it would be possible to verify the accurate efficacy of the recombinant protein Lin28-30Kc19 on hiPSC production. Moreover, understanding the mechanisms of the protein-based reprogramming by Lin28-30Kc19 enables more comprehensive applications of this protein. There remained more things to do, however considering these effects of the Lin28-30Kc19 protein, we believe that it would be a useful substance that can efficiently supply iPSCs necessary for the medical and pharmaceutical industry and biotechnology research areas. It can also be applied to the binding of the 30Kc19 protein to other significant reprogramming factors to produce protein-based human somatic cell-derived iPSCs.

## Conclusion

In summary, we have demonstrated an innovative approach to improve the efficiency of human-induced pluripotent stem cell (hiPSC) generation in respect of AP-positive colonies, *via* protein-based delivery. This was achieved by using an important transcriptional factor, Lin28, fusing with a cell-penetrating protein, 30Kc19, in the form of a recombinant fusion protein. Mass production of a soluble form of the Lin28-30Kc19 protein was achieved, and thus treated to human dermal fibroblasts (HDFs), in addition to four transcriptional factors (Oct4, Sox2, c-Myc, and Klf4), which were delivered through a retrovirus. We efficiently expressed and purified the fusion protein in *E. coli*, and then characterized the *in vitro* properties of the Lin28-30Kc19 protein. By optimizing the concentration of the treated Lin28-30Kc19 protein and the interval for the protein treatment, the efficacy of AP-positive hiPSC colony generation was increased compared with the control group (only four factors). We emphasize the importance of recombinant protein production in the fused form with 30Kc19 in order to improve the solubility and stability of the protein. And, we also assert the significance of the Lin28-30Kc19 protein addition on the efficiency of reprogramming HDFs into hiPSCs. We believe that the research findings reported in this manuscript represent a significant advancement in the field of protein-based reprogramming research.

## Data Availability

The raw data generated for this study are available on request to the corresponding author.
